# Phase II trial of blood–brain barrier permeable peptide-paclitaxel conjugate ANG1005 in patients with recurrent high-grade glioma

**DOI:** 10.1093/noajnl/vdae186

**Published:** 2024-12-14

**Authors:** Crismita Dmello, Andrew Brenner, David Piccioni, Patrick Y Wen, Jan Drappatz, Maciej Mrugala, Lionel D Lewis, David Schiff, Camilo E Fadul, Marc Chamberlain, Santosh Kesari, Manmeet Ahluwalia, Debora Ghosh, Adam M Sonabend, Priya Kumthekar

**Affiliations:** Northwestern Medicine Malnati Brain Tumor Institute of the Lurie Comprehensive Cancer Center, Feinberg School of Medicine, Chicago, Illinois, USA; Department of Neurological Surgery, Northwestern University, Feinberg School of Medicine, Chicago, Illinois, USA; Cancer Therapy and Research Center, University of Texas Health Science Center, San Antonio, Texas, USA; Department of Neurosciences, UC San Diego Moores Cancer Center, La Jolla, California, USA; Center For Neuro-Oncology, Dana Farber Cancer Institute, Boston, Massachusetts, USA; Department of Neurology, UPMC Hillman Cancer Center, Pittsburgh, Pennsylvania, USA; Fred Hutchinson Cancer Center, University of Washington Medical Center Seattle, Washington, USA; Department of Medicine, Dartmouth-Hitchcock Medical Center Lebanon, New Hampshire, USA; Emily Couric Clinical Cancer Center, University of Virginia, Charlottesville, Virginia, USA; Division of Neuro-Oncology, Department of Neurology, University of Virginia, Charlottesville, Virginia, USA; Lantern Pharma, Dallas, Texas, USA; Department of Translational Neurosciences, Pacific Neuroscience Institute and Saint John’s Cancer Institute at Providence Saint John’s Health Center, Santa Monica, California, USA; Department of Medical Oncology, Miami Cancer Institute, Miami, Florida, USA; Department of Ophthalmology, Boston Children’s Hospital, Harvard Medical Center, Boston, Massachusetts, USA; Northwestern Medicine Malnati Brain Tumor Institute of the Lurie Comprehensive Cancer Center, Feinberg School of Medicine, Chicago, Illinois, USA; Department of Neurological Surgery, Northwestern University, Feinberg School of Medicine, Chicago, Illinois, USA; Department of Neurology, Northwestern University, Feinberg School of Medicine, Chicago, Illinois, USA; Northwestern Medicine Malnati Brain Tumor Institute of the Lurie Comprehensive Cancer Center, Feinberg School of Medicine, Chicago, Illinois, USA

**Keywords:** ANG1005, high-grade glioma, paclitaxel, phase II clinical trial

## Abstract

**Background:**

This study is a phase II clinical trial to evaluate the efficacy, safety, and tolerability of the blood–brain barrier (BBB) permeable peptide-paclitaxel conjugate ANG1005 in patients with recurrent high-grade glioma (HGG) (NCT01967810).

**Methods:**

Seventy-three patients were enrolled in 3 separate arms-recurrent glioblastoma (GBM) (Arm 1), bevacizumab refractory GBM (Arm 2), and grade 3 anaplastic gliomas (AGs) (Arm 3). The study was started in October 2013, and the data were locked on September 29, 2017. Safety was evaluated for all three arms (*n* = 73), and the primary endpoint for Arms 1 and 3 was objective response rate (ORR), and Arm 2 primary endpoint was progression-free survival rate at 3 months (PFS3).

**Results:**

Overall, the safety of ANG1005 was found to be consistent with a taxane toxicity profile. Otherwise, the primary efficacy endpoints of ORR and PFS were not met. The most common adverse events (AEs) were hematologic (32.9%), alopecia (31.5%), and fatigue (30.1%). The median PFS was 1.4 months (95% CI: 1.4, 2.1) and similar across all the treatment arms. The median overall survival was 13.4 months (95% CI: 3.4, 14.6) in Arm 1, 5.8 months (95% CI: 1.9, 9.7) in Arm 2, and 18.2 months (95% CI: 10.7, 35.3) in Arm 3.

**Conclusion:**

A dose of 600 mg/m^2^ was determined to be safe in this study. However, the primary efficacy endpoint was not met in the NCT01967810-ANG1005 trial, and no further studies are planned in the glioma setting with this compound.

## Lay Summary

High-grade gliomas (HGGs) are aggressive brain cancer. ANG1005 is a new chemotherapy drug that was designed to specifically pass through a natural barrier that protects the brain from drugs. The authors of this study wanted to know whether this drug was safe and helpful for patients with HGGs. To do this, they treated 73 patients with HGGs in a clinical trial. The authors concluded that ANG1005 was overall safe at a dose of 600 mg/m². Common side effects were low blood counts, hair loss, and fatigue. However, ANG1005 did not seem to reduce the tumor size or slow down its growth.

**Key Points**

1. A dose of 600 mg/m^2^ was determined to be safe in this study.
2. The NCT01967810-ANG1005 trial did not demonstrate significant efficacy in the heavily pretreated recurrent HGG patients.

**Importance of the Study**

This original research is the prospective study of patients with recurrent high-grade glioma (HGG) treated with blood–brain barrier (BBB) permeable peptide-paclitaxel conjugate ANG1005. In this Phase II clinical trial, the efficacy, safety, and tolerability profile of ANG1005 was determined. ANG1005 was shown to be safe in patients with recurrent HGG, including in patients with GBM who were bevacizumab refractory. However, the NCT01967810 clinical trial did not demonstrate significant efficacy in the heavily pretreated recurrent HGG patients. This study demonstrates the potential impact of leveraging peptide-drug conjugates to cross the BBB. Moreover, if larger clinical trials using paclitaxel drug formulation were to be conducted, it would be crucial to use predictive biomarker to prospectively predict response in these patients.

Effective drug therapy for central nervous system (CNS) malignancies has traditionally been elusive due in part to the limited blood–brain barrier (BBB) penetration of most chemotherapeutic agents.^1^ High-grade gliomas (HGGs) are the most aggressive primary brain tumors, for which the treatment options are limited.^2–4^ The mainstay of their treatment remains a combination of radiotherapy and chemotherapy with temozolomide.^5^ However, no single standard of care exists for recurrent HGG given the lack of demonstrated efficacy of innumerable agents.^6,7^

Paclitaxel is a chemotherapeutic agent used to treat a variety of systemic cancers that act by stabilizing microtubules, causing inhibition of cell proliferation and promotion of cell apoptosis.^8^ While in vitro studies have shown activity against HGG, it has been unsuccessful in human studies^9^ largely because of its inability to penetrate the BBB.^10^ ANG1005 (paclitaxel trevatide) is a novel drug conjugate, consisting of three molecules of paclitaxel linked to the amino acid peptide Angiopep-2. Angiopep-2 targets the low-density lipoprotein receptor-related protein (LRP-1), which is readily expressed on the capillary endothelial cells of the BBB and upregulated in HGG.^11,12^ The combination of paclitaxel with Angiopep-2 enhances the drug delivery of paclitaxel into the brain parenchyma.^13^

A phase I dose-escalation study of ANG1005 revealed that the drug was detected in recurrent gliomas in therapeutic concentrations, demonstrating transport across BBB and successful tumor penetration. The overall safety profile was favorable and consistent with the known side effect profile of unconjugated paclitaxel. The results of the Phase I study of ANG 1005 in breast cancer patients with leptomeningeal carcinomatosis and recurrent brain metastases demonstrated transport of ANG1005 across the BBB and achievement of tumor penetration at therapeutic concentrations in recurrent glioma tumors following a single intravenous infusion.^14^ In another phase I testing of ANG1005 in patients with brain metastases from breast tumors and other advanced solid tumors, 22% (4/18) of the patients were observed with an overall partial response (PR) and 56% (10/18) with stable disease (SD) at doses ≥ 420 mg/m^2^ for CNS response. 25% (4/16) of the patients were observed with an overall PR and 44% (7/16) with SD at doses ≥ 420 mg/m^2^ for non-CNS peripheral metastases response.^15^

The objective of this phase II study (predated 2013-2017) was to evaluate the objective response, efficacy, safety, and tolerability of ANG1005 administered to individuals with recurrent glioblastoma (GBM) (Arm 1), bevacizumab refractory GBM (Arm 2), and grade 3 glioma (Arm 3).

## Patients and Methods

### Study Design

This Phase II, multicenter, open-label study aimed to evaluate the efficacy, safety, and tolerability of ANG1005 in patients with recurrent HGG. The protocol was reviewed and approved by the institutional review board of each participating center. All patients gave written informed consent for trial participation. According to 2007 WHO classification used in this study, HGG included grade 3 AGs such as anaplastic astrocytomas (AAs), anaplastic oligodendrogliomas (AOs), and anaplastic oligoastrocytomas (AOAs), as well as grade 4 GBM.^16^ Eligibility criteria included the following: adults ≥ 18 years of age; histologically confirmed GBM or GBM variants, including gliosarcoma, GBM with oligodendroglioma components; anaplastic World Health Organization (WHO) Grade III gliomas such as astrocytoma and oligodendroglioma (based on WHO 2007^16^ given the study’s enrollment years); and ≥ 1 radiologically confirmed and bidimensionally measurable brain lesion as defined by the RANO criteria^17^ that was previously untreated by stereotactic radiosurgery. Additionally, a Karnofsky Performance Scale (KPS) score ≥ 80, neurologic stability with patients receiving stable doses of corticosteroids and anticonvulsants, and normal hepatic, renal, and hematologic function with ≥ 3 months of expected survival were requirements for inclusion. In patients with gliomas refractory to bevacizumab, radiological confirmation of tumor progression during bevacizumab therapy was necessary.

Exclusion criteria included the following: patients with > 3 relapses; prior treatment before study entry (within 4 weeks or 5 half-lives, whichever was shorter of Cycle 1, Day 1), including radiotherapy (within 3 months), surgery, chemotherapy, investigational drugs, nitrosoureas, biologic and immunotherapy (excluding bevacizumab for Arm 2), exposure to P450 CYP 3A4 or 2C8 enzyme-inducing anticonvulsant drugs (within 2 weeks), or previous treatment with ANG1005/GRN1005, taxanes, or bevacizumab within 4 weeks of treatment initiation in patients with recurrent WHO Grade 3 gliomas. Evidence of severe intracranial hemorrhage, peripheral neuropathy grade ≥ 2, evidence of severe or uncontrolled disease, signs of infection, including hepatitis B, C, or human immunodeficiency virus (HIV), inadequate bone marrow reserve, and known severe hypersensitivity reactions or allergies to paclitaxel or its components were reasons for exclusion from the study.

Gadolinium-enhanced magnetic resonance imaging of the brain was used to evaluate disease extent beginning at baseline and continued every two treatment cycles (ie, every 6 ± 2 weeks) until disease progression or unacceptable treatment toxicity. Upon partial or complete response (CR) of tumor to ANG1005 treatment, a confirmatory MRI was conducted approximately 6 weeks to confirm response. The Response Assessment in Neuro-Oncology (RANO) was utilized for two-dimensional intracranial tumor assessment.^17^

### Treatments Administered

The initial starting dose of ANG1005 was 650 mg/m^2^ as an IV infusion at a rate of 8.0–8.5 mL/minute on Day 1 of each 21-day cycle (± 3 days). Following the dosing of the first 4 patients, the starting dose was decreased to 600 mg/m^2^ to improve tolerability while maintaining efficacy. Subsequently, a total of 69 patients were enrolled at 600 mg/m^2^ as a starting dose. Dose reductions or delays were allowed if toxicity was observed. Patients were monitored for a minimum of 1 h after the completion of each infusion. Treatment for infusion-related reactions and management of ANG1005 dosing was based on the severity and resolution of the event and managed according to the protocol. ANG1005 was administered for up to maximum of 1 year or until disease progression or AEs that were not tolerated. Further treatment of patients who remained eligible beyond the 1-year maximum treatment period was considered on a case-by-case basis and included only a single patient (Arm 3) who responded to treatment (PR, followed by CR) and received 25 cycles of ANG1005 over approximately 18 months.

### Dose Modifications and Delays

Dose reductions and delays for ANG1005 in response to hematological and other AEs were permitted. In response to hematological events and AEs, the dose of ANG1005 was reduced by one dose level (ie, from 600 to 550 mg/m^2^, from 550 to 470 mg/m^2^, or from 470 to 400 mg/m^2^), per protocol. Dose reductions were mandatory for all patients who experienced any (life-threatening) Grade 4 toxicity, and for selected Grade 3 toxicities, such as Grade 3 pneumonitis, Grade 3 peripheral neuropathy or CNS toxicity, or Grade 3 cardiac adverse events (AEs). Increases in ANG1005 dose were not allowed once the dose had been reduced. ANG1005 treatment could be delayed for up to 3 weeks, after which the patient was considered for withdrawal from the study.

### Evaluation of Efﬁcacy

The primary objectives of the study were to determine (1) the objective response rate (ORR) in bevacizumab-naïve recurrent GBM patients (Arm 1), (2) progression-free survival rate at 3 months (PFS3) in the bevacizumab-refractory recurrent GBM patients (Arm 2), and (3) ORR in recurrent AG patients (Arm 3). The arms were predefined. Bevacizumab was not continued in bevacizumab-refractory patients.

The secondary objectives of this study were to determine (1) the ORR in Arm 2; (2) number of patients without progression at 3, 6, and 12 months in Arms 1 and 3; (3) number of patients without progression at 6 and 12 months in Arm 2; (4) median progression-free survival in each arm; (5) median duration of response in each arm; (6) median overall survival (OS) in each arm; (7) safety and tolerability; and (8) plasma pharmacokinetic profile.

The exploratory objectives of this study were (1) immunogenicity assessment of ANG1005; (2) ORR, PFS3, and PFS6, as determined by an Independent Radiology Reviewer; (3) neurocognitive function changes; and (4) molecular correlation of clinical outcome with LRP-1 receptor status, protease levels, and potential glioma biomarkers from tumor specimens when available.

### Neurocognitive Testing

Neurocognitive function testing was administered prior to ANG1005 infusion on Day 1 (−7 days) of Cycles 1, 5, 9, and 17 and at the end of the treatment visit. The end of treatment assessment was only performed if no neurocognitive assessment was done within 30 (± 3) days. Information regarding the patient’s demographic background was collected. Neurocognitive test administrators were trained and provided with a manual to ensure standardized test administration. Neurocognitive test results were evaluated and read centrally. Three domains of neurocognitive function were measured as exploratory endpoints in this study: memory, visuomotor scanning speed (eg, complex visual scanning with a motor component), and executive function (eg, mental flexibility). The neurocognitive battery included the following tests: Hopkins Verbal Learning Tests—Revised (HVLT-R Total Recall, Delayed Recall, Delayed Recognition), Trail Making Test Part A and Part B^18^ (TMBTA, TMBTB), and Controlled Oral Word Association (COWA).^19^

### Archived Brain Tumor Tissue

An optional archival brain tumor tissue collection was requested for exploratory marker analyses, if available. Of the 73 patients enrolled in the trial, archival tumor samples from 26 patients were obtained.

### Statistical Analysis

#### *Efficacy analysis*.—

ORR was defined as the proportion of patients with a CR or PR. The assessment was made according to RANO criteria based on the investigator’s assessment.^17^ The ORR was the primary endpoint for Arms 1 and 3 and a secondary endpoint for Arm 2. ORR for each arm was calculated, and a 2-sided 95% confidence interval was provided using the exact method based on the binomial distribution. PFS was defined as the time from study enrollment to documented disease progression as determined by the local investigator, clear clinical progression in the absence of a brain MRI determination of progression, or death from any cause, whichever occurred first. PFS was the primary endpoint for Arm 2 and a secondary endpoint for Arms 1 and 3. Patients with a clinical determination of progression underwent an MRI assessment. If a clinical determination of progression for a patient was confirmed by an MRI, the date of the MRI was considered as the progression date for that patient. The data for patients who were still alive and free from disease progression at the time of data cutoff were administratively censored on the last assessment (or, if no post-baseline tumor assessment, at the time of first dose plus 1 day). Data for patients who were lost to follow-up prior to documented disease progression were censored at the last disease assessment date when the patient was known to be disease progression-free. Kaplan–Meier methodology was used to estimate the 3-month PFS rate, and 95% confidence intervals for PFS rate were computed using Greenwood’s formula by arms. In addition, the median PFS was calculated for each arm.

#### Safety analysis.—

Evaluation of safety included the analysis of AEs and the analysis of laboratory and vital signs data using the safety population. Verbatim descriptions of AEs were mapped according to the Medical Dictionary for Regulatory Activities (MedDRA) v17.0 or later thesaurus terms and graded according to the NCI CTCAE v4.0. Incidence of AEs, severe AEs, serious AEs (SAEs), non-serious AEs leading to study treatment discontinuation, and study drug-related AEs was summarized by system organ class (SOC) and preferred terms according to MedDRA as well as severity per NCI CTCAE grade. Laboratory data was analyzed with shift tables and summaries of change from baseline to maximum post-treatment value based on local lab normal ranges. Change from baseline in vital signs were summarized. Neurocognitive function test data was analyzed by descriptive statistics (mean, SD and median) and change in neurocognitive function from baseline to each follow-up time point was categorized as improved, stable, or declined based on the reliable change index (RCI) for each test.

#### Sample size calculation.—

The primary objective of the bevacizumab-naive in recurrent GBM arm (Arm 1) was to determine the efficacy of ANG1005 as measured by ORR. This arm was planned to enroll enough patients (*N* = 37) to discriminate between a 5% and 20% ORR with 90% power and a 1-sided type I error rate of 10%. The primary objective of the bevacizumab-refractory in recurrent GBM arm (Arm 2) was to determine the efficacy of ANG1005 as measured by PFS at 3 months (PFS3), which is a commonly reported primary endpoint for comparable studies in this patient population. This arm was planned to enroll enough patients (*N* = 22) to discriminate between a PFS3 of 10% versus 35% with 93% power and a 1-sided type I error rate of less than 10%. The primary objective of the recurrent AG arm (Arm 3) was to determine the efficacy of ANG1005 as measured by ORR. This arm was planned to enroll enough patients (*N* = 24) to discriminate between a 5% and 25% ORR with 90% power and a 1-sided type I error rate of 10%. It was estimated that up to 90 patients would be enrolled in this study to reach the number of evaluable patients in each arm. This estimate was based on a maximum of 32 evaluable patients in Arm 1, 22 evaluable patients in Arm 2, and 20 evaluable patients in Arm 3. In total, 73 patients were enrolled in the study.

## Results

### Patients and Treatments

This phase II study enrolled 73 patients with recurrent HGG and its duration was 4 years (study start date: October 25, 2013 and locked data available: September 29, 2017). All patients enrolled in the study (*n* = 73) received ANG1005 and were evaluated according to three arms defined by disease type and prior treatment. Arm 1 (*n* = 24) comprised patients with recurrent GBM without previous exposure to bevacizumab treatment, while Arm 2 (*n* = 16) consisted of patients whose disease was refractory to bevacizumab treatment. Arm 3 (*n* = 33) evaluated patients with WHO Grade III AG naive to bevacizumab. Given the known worsened OS in bevacizumab refractory patient in comparison to bevacizumab naive patients, cohorts on this study were decided accordingly. This was done with the intention to appropriately compare outcomes as opposed to inherent biologic activity of ANG1005 with or without bevacizumab.

**Figure 1.**
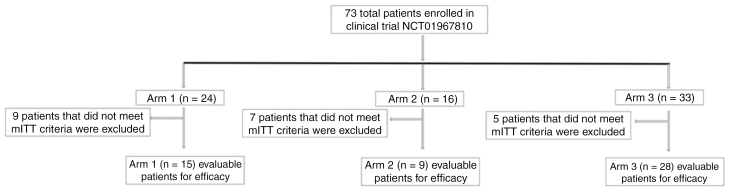
Study schema (CONSORT diagram) of ANG1005 NCT01967810 clinical trial. All the patients enrolled to receive ANG1005 were evaluated in three separate arms according to the type of disease and disease state for efficacy.

All 73 enrolled patients were included in the safety population, with 52 patients included in the modified Intent to Treat (mITT). By arm, 24 subjects enrolled in Arm 1, 16 subjects in Arm 2, and 33 subjects in Arm 3 (Figure 1). Three subjects (all in Arm 3) completed the 1-year study treatment. Overall, treatment was discontinued most frequently due to disease progression (41 subjects or 56.2%), patient withdrawal from treatment (16 subjects [21.9%], and AEs (7 subjects [9.6%]). The less common reasons for discontinuation were investigator and sponsor decisions to withdraw (1 subject [1.4%]), death (4 subjects [5.5%]), and other reasons (1 subject [1.4%]). Distribution of primary reasons for study treatment discontinuation. In the Swimmer’s plots, we have indicated the reasons for treatment discontinuation and compared them to the PFS. Supplementary Figure 1 displays time points of reasons for treatment discontinuation in relation to PFS. For all treatment arms, the primary reason for study termination was death (53 subjects overall [72.6%]).

Median age of Arm 1 was 53.5 years (range, 26–75), Arm 2 was 55.0 years (range, 42–79), and Arm 3 was 45.0 years (range, 18–75), as shown in Table 1. There were no relevant differences across the treatment arms for any of the demographic and baseline characteristics reported. Male patients comprise 66.7% (*n* = 16) in the Arm 1, 62.5% (*n* = 10) in the Arm 2, and 60.6% (*n* = 20) in the Arm 3. The median age of all patients in the study was 51.0 years, with a range from 18 to 79 years. Most subjects were male (46 subjects [63.0%]). At treatment onset, all patients had KPS values ≥ 70. Two patients in Arm 3 had KPS not meeting criteria on Cycle 1 Day 1. The baseline KPS distribution in 3 arms is shown in Table 1. Information about MGMT methylation (which confers prognostic significance) was not routinely collected for these patients. IDH status information was available on 16.4% (*n* = 12) of 73 patients with 9.5% (*n* = 7) IDH mutant patients, and 6.8% (*n* = 5) IDH wild-type patients. Supplementary Table 1 lists the pathologic diagnosis, grade, IDH status, PFS, OS, etc. for all the 73 patients.

**Table 1.** Baseline Patient Characteristics and Oncology History

Baseline Characteristics	Arm 1 (*N* = 24)	Arm 2 (*N* = 16)	Arm 3 (*N* = 33)	Overall (*N* = 73)
*N*	24	16	33	73
Age (years) mean (SD)	54.6 (11.65)	55.8 (10.75)	45.6 (14.82)	50.8 (13.70)
Age (years) median	53.5	55.0	45.0	51.0
Age (years) min, max	26, 75	42, 79	18, 75	18, 79
Sex, *n* (%)				
Male	16 (66.7)	10 (62.5)	20 (60.6)	46 (63.0)
Female	8 (33.3)	6 (37.5)	13 (39.4)	27 (37.0)
KPS, *n* (%)				
60	0	1 (6.3)	1 (3.0)	2 (2.7)
70	3 (12.5)	4 (25.0)	5 (15.2)	12 (16.4)
80	10 (41.7)	6 (37.5)	11 (33.3)	27 (37.0)
90	8 (33.3)	5 (31.3)	11 (33.3)	24 (32.9)
100	3 (12.5)	0	5 (15.2)	8 (11.0)
Prior therapies Median (range)	5 (3–9)	5 (3–7)	4 (1–11)	5 (1–11)
Tumor type, *n* (%)				
Glioblastoma	19 (79.2)	16 (100)	1 (3.0)	36 (49.3)
Gliosarcoma	0	0	0	0
Glioblastoma with oligodendroglial component	2 (8.3)	0	0	2 (2.7)
Anaplastic astrocytoma	1 (4.2)	0	16 (48.5)	17 (23.3)
Anaplastic oligodendroglioma	0	0	12 (36.4)	12 (16.4)
Anaplastic oligoastrocytoma	0	0	3 (9.1)	3 (4.1)
Other	2 (8.3)	0	1 (3.0)	3 (4.1)
Grade *n* (%)				
WHO Stage III	0	0	30 (90.9)	30 (41.0)
WHO Stage IV	23 (95.8)	16 (100)	0	39 (53.4)
Other	1 (4.2)	0	3 (9.1)	4 (5.5)
Prior bevacizumab, *n* (%)	0	16 (100)	6 (18.2)	21 (28.8)

Abbreviation: KPS, Karnofsky Performance Scale.

All patients had been treated prior to the study with a median of 5 therapies (range from 1 to 11), and the mean (SD) years from initial diagnosis was 3.2 (3.1) years. Overall, the most common prior therapies during the recurrent stage were chemotherapy (71 patients [97.3%]), surgery (65 patients [89.0%]) and radiotherapy (42 patients [57.5%]). Median prior therapies received by Arm 1 patients was 5 (range: 3, 9), Arm 2 patients received 5 (range: 3, 7), and Arm 3 patients received 4 (range: 1, 11). The Arm 1 patients comprise 79.2% (*n* = 19) diagnosed with GBM, 8.3% (*n* = 2) diagnosed with GBM with oligodendroglial component, 4.2% (*n* = 1) diagnosed with AA and 8.3% (*n* = 2) placed in other tumor type category (based on the WHO 2007 CNS tumor classification^16^). The Arm 2 patients comprise 100% (*n* = 16) diagnosed with GBM. The Arm 3 patients comprise 3% (*n* = 1) diagnosed with GBM, 48.5% (*n* = 16) diagnosed with AA, 36.4% (*n* = 12) diagnosed with AO, 4.1% (*n* = 3) diagnosed with AOA, and 4.1% (*n* = 3) placed in other tumor type category. The distribution of WHO stages and prior bevacizumab treatment is shown in Table 1.

### Safety

All patients experienced at least one AE. The most commonly reported AEs were nervous system disorders including peripheral neuropathy (57 patients [78.1%]), general disorders and administration site reactions (50 patients [68.5%]), gastrointestinal disorders (45 patients [61.6%]), and skin and subcutaneous disorders (44 patients [60.3%]). By SOC, the most commonly reported related AEs were general disorders and administration site reactions (42 patients [57.5%]), blood and lymphatic system disorders (37 patients [50.7%]), skin and subcutaneous tissue disorders (35 patients [47.9%]), and gastrointestinal disorders (34 patients [46.6%]). Over the course of the study, 3 AEs led to death: 2 events in Arm 2 (septic shock and pulmonary embolism) and one event in Arm 3 (hydrocephalus). Of the 3 AEs, only the AE of septic shock was considered potentially related to treatment with ANG1005. SAEs were recorded for 33 patients (45.2%). Nineteen patients (26.0%), 9 patients (12.3%) and 3 patients (4.1%) experienced a Grade 3, 4, or 5 SAE, respectively. SAEs considered related to ANG1005 were recorded for 21 patients (28.8%), as shown in Table 2.

**Table 2.** Severe Adverse Events (SAE) in Evaluable Patients (*N* = 73)

All serious adverse events	Overall (*N* = 73) *n* (%)
Patients reporting at least one event	33 (45.2)
General disorders and administration site conditions	10 (13.7)
Nervous system disorders	10 (13.7)
Blood and lymphatic system disorders	8 (11.0)
Infections and infestations	7 (9.6)
Gastrointestinal disorders	3 (4.1)
Cardiac disorders	2 (2.7)
Respiratory, thoracic, and mediastinal disorders	2 (2.7)
Immune system disorders	1 (1.4)
Injury, poisoning, and procedural complications	1 (1.4)
Investigations	1 (1.4)
Metabolism and nutrition disorders	1 (1.4)
Musculoskeletal and connective tissue disorders	1 (1.4)
Psychiatric disorders	1 (1.4)
Skin and subcutaneous tissue disorders	1 (1.4)

Serious adverse events related to ANG1005	Overall (*N* = 73) *n* (%)
Patients reporting at least one related SAE	21 (28.8)
General disorders and administration site conditions	9 (12.3)
Blood and lymphatic system disorders	8 (11.0)
Infections and infestations	4 (5.5)
Gastrointestinal disorders	2 (2.7)
Immune system disorders	1 (1.4)
Investigations	1 (1.4)
Metabolism and nutrition disorders	1 (1.4)
Musculoskeletal and connective tissue disorders	1 (1.4)

### Efficacy Analysis

Of the 73 patients enrolled, 52 were considered evaluable for efficacy per protocol (ie, the mITT population). The mITT population (*n* = 52) included patients treated with ANG1005 who had a clinical evaluation and/or a postdose scan at ≥ 4 weeks from Cycle 1, Day 1. The 21 patients were excluded from mITT on one or both grounds. The mITT analysis set was the primary efficacy analysis population and was used in the efficacy analyses (Figure 1). The ORR as assessed by the investigator, progression-free survival, and OS is presented in Table 3.

**Table 3.** Overview of Selected Efficacy Parameters (mITT Population)

ANG1005 Parameter	Arm	Stable	Improved	Declined
HVLT-R A Total	Arm 1	3/5 (60.0%)	0/5 (0%)	2/5 (40.0%)
	Arm 2	3/4 (75.0%)	0/4 (0%)	1/4 (25.0%)
	Arm 3	11/13 (84.6%)	1/13 (7.7%)	1/13 (7.7%)
	Total	17/22 (77.3%)	1/22 (4.5%)	4/22 (18.2%)
HVLT-R B	Arm 1	4/5 (80.0%)	0/5 (0%)	1/5 (20.0%)
	Arm 2	1/4 (25.0%)	1/4 (25.0%)	2/4 (50.0%)
	Arm 3	8/13 (61.5%)	1/13 (7.7%)	4/13 (30.8%)
	Total	13/22 (59.1%)	2/22 (9.1%)	7/22 (31.8%)
HVLT-R C	Arm 1	4/5 (80%)	0/5 (0%)	1/5 (20.0%)
	Arm 2	3/4 (75%)	0/4 (0%)	1/4 (25.0%)
	Arm 3	5/13 (38.5%)	5/13 (38.5%)	3/13 (23.1%)
	Total	12/22 (54.5%)	5/22 (22.7%)	5/22 (22.7%)
TMTA	Arm 1	4/5 (80.0%)	0/5 (0%)	1/5 (20.0%)
	Arm 2	3/4 (75.0%)	0/4 (0%)	1/4 (25.0%)
	Arm 3	7/11 (63.6%)	1/11 (9.1%)	3/11 (27.3%)
	Total	14/20 (70.0%)	1/20 (5.0%)	5/20 (25.0%)
TMTB	Arm 1	2/5 (40.0%)	0/5 (0%)	3/5 (60.0%)
	Arm 2	3/4 (75.0%)	0/4 (0%)	1/4 (25.0%)
	Arm 3	9/12 (75.0%)	2/12 (16.7%)	1/12 (8.3%)
	Total	14/21 (66.7%)	2/21 (9.5%)	5/21 (23.8%)
COWA Total	Arm 1	5/5 (100.0%)	0/5 (0%)	0/5 (0%)
	Arm 2	1/4 (25.0%)	1/4 (25.0%)	2/4 (50.0%)
	Arm 3	13/13 (100.0%)	0/13 (0%)	0/13 (0%)
	Total	19/22 (86.4%)	1/22 (4.5%)	2/22 (9.1%)

Abbreviation: COWA, Controlled Oral Word Association; HVLT, Hopkins Verbal Learning Test; mITT, modified Intent to Treat; TMT, Trail Making Test.

**Figure 2.**
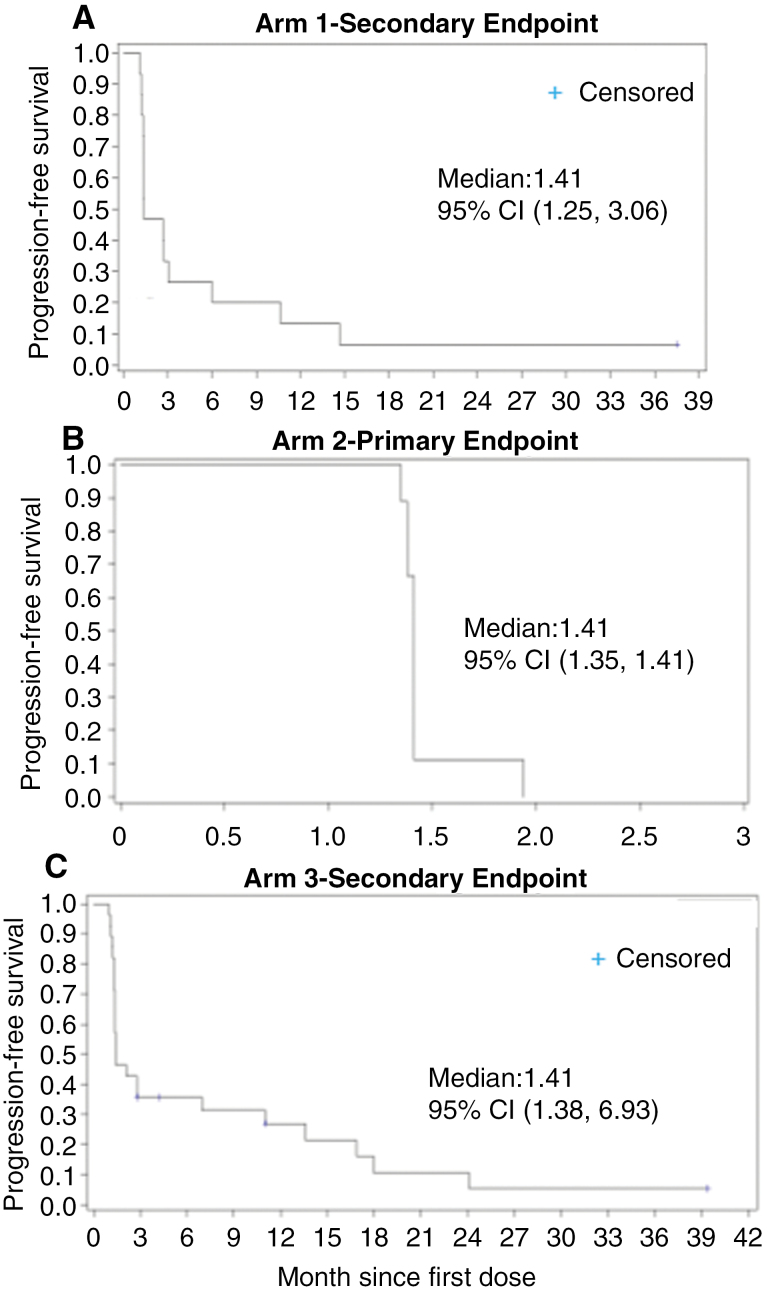
PFS outcomes of different arms in ANG1005 clinical trial. Kaplan–Meier estimates of PFS in Arm 1 (A), Arm 2 (B), and Arm 3 (C).

**Figure 3.**
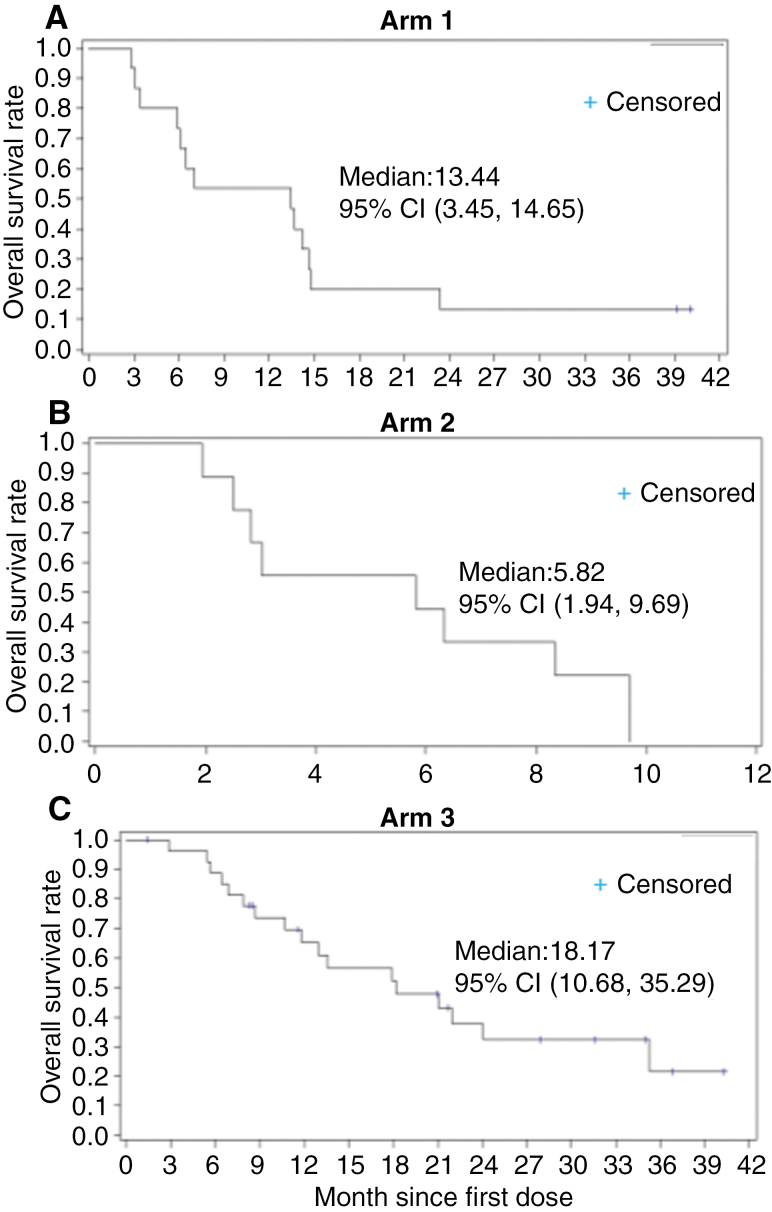
OS outcomes of different arms in ANG1005 clinical trial. Kaplan–Meier estimates of OS in Arm 1 (A), Arm 2 (B), and Arm 3 (C).

**Figure 4.**
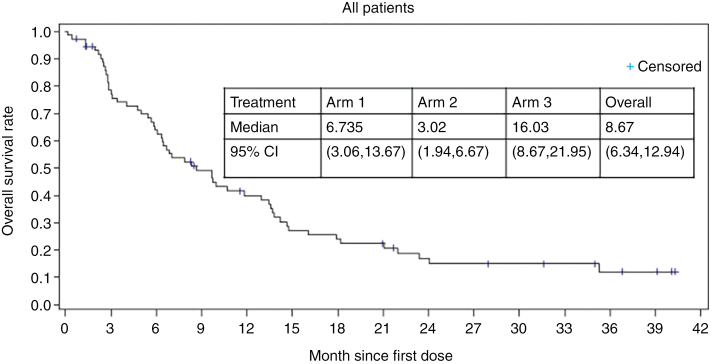
OS outcomes of all the patients in ANG1005 clinical trial. Kaplan–Meier estimates of OS for all the patients (*N* = 73).

(i) ORR: A complete or PR was recorded for 3 subjects, with an overall ORR of 5.8% (95% CI: 1.2, 15.9). Of the three responders: 1 CR (3.6%) was recorded in Arm 3 (primary endpoint), 2 PRs (13.3%) were recorded in Arm 1 (primary endpoint), and none in Arm 2 (secondary endpoint). Overall, SD was recorded for 21 subjects (40.4%), and progressive disease was recorded for 28 subjects (53.8%). Distribution of SD across the arms were 5 subjects (33.3%) in Arm 1, 2 subjects (22.2%) in Arm 2, and 14 subjects (50.0%) in Arm 3. Distribution of progressive disease across the arms was 8 subjects (53.3%) in Arm 1, 7 subjects (77.8%) in Arm 2, and 13 subjects (46.4%) in Arm 3. The clinical benefit rate (defined as SD or better) was seen in all arms with 46.6% patients with disease control in Arm 1, 22.2% in Arm 2, and 53.6% in Arm 3.
(ii) Duration of Response: Of the 3 responders (2 in Arm 1 and 1 in Arm 3) in the efficacy evaluable population, the median duration of response was 13.4 months (95% CI: 2.1, NA). The median duration of response in Arm 1 was 7.75 months (95% CI: 2.1, 13.4) and was not reached in Arm 3. The duration rate was identical at each of the 3, 6, and 12-month time points (Arm 1: 50.0% [95% CI: 0.6, 91.0], Arm 3: 100% [95% CI: 100,100] and overall: 66.7% [95% CI: 5.4, 94.5]). Duration of response was defined as the time from the first tumor assessment demonstrating objective response to the time of disease progression determined by MRI, clear clinical progression in the absence of an MRI determination of progression, or death from any cause, whichever occurred first.
(iii) Progression-free survival: PFS was assessed by the investigator. Most subjects progressed or died (47 or 90.4%), with five subjects being censored (1 subject [6.7%] in Arm 1 and 4 subjects [14.3%] in Arm 3). All subjects in Arm 2 (primary endpoint) progressed or died. Overall median PFS was 1.4 months (95% CI: 1.4, 2.1) across all 3 arms. The median PFS was similar across all treatment arms. In addition, the PFS (secondary endpoints) rates were 3 months: 33.3% (95% CI: 12.2, 56.4) in Arm 1 and 35.7% (95% CI: 18.9, 53.0) in Arm 3, 6 months: 26.7% (95% CI: 8.3, 49.6) in Arm 1 and 35.7% (95% CI: 18.9, 53.0) in Arm 3, and 12 months: 13.3% (95% CI: 2.2, 34.6) in Arm 1 and 26.8% (95% CI: 11.8, 44.4) in Arm 3. In Arm 2, PFS rates were 0 at each time point (Figure 2A–C).
(iv) OS: OS was analyzed from both the mITT population and the safety population. For the mITT population (*n* = 52), the overall median OS was 12.94 months (95% CI: 7.03, 14.78). Median OS was 13.4 months (95% CI: 3.4, 14.6) in Arm 1, 5.8 months (95% CI: 1.9, 9.7) in Arm 2, and 18.2 months (95% CI: 10.7, 35.3) in Arm 3 (Figure 3A–C). For the safety population (*n* = 73), overall median OS was 8.67 months (95% CI: 6.34, 12.94). Median OS was 6.74 months (95% CI: 3.06, 13.67) in Arm 1, 3.02 months (95% CI: 1.94, 6.67) in Arm 2, and 16.03 months (95% CI: 8.67, 21.95) in Arm 3 (Figure 4).

### Neurocognitive Function Evaluations

Based on the limited neurocognitive function data, stratification per arm was only possible at the end of treatment (EOT) visit although sample size remained small for Arm 1 (*n* = 5) and Arm 2 (*n* = 4) to moderate for Arm 3 (*n* = 13). Based on RCI, neurocognitive function was stable or improved in 40–100% of patients in Arm 1, 50–75% in Arm 2, and 69–100% in Arm 3, across the battery of tests (Table 4). Based on the Composite Triple Beat (CTB) score, Arm 3 had more patients with stable (67%) or improved (8%) neurocognitive function, as compared to the other two arms where either 60% (Arm 1) or 50% (Arm 2) of the patients showed stable neurocognitive function, and there were no patients with improved function in these two arms. Decline in neurocognitive function was also seen less frequently in Arm 3 (25%), as compared to Arm 1 (40%) and Arm 2 (50%), based on the CTB Composite score. Overall, stable or improved neurocognitive function was seen more frequently than a decline following ANG1005 treatment in a small sample size of patients.

**Table 4.** Neurocognitive Function Test RCI Change Summary per Arm-End of Treatment Visit (EOT)

ANG1005 Parameter	Arm	Stable	Improved	Declined
HVLT-R A Total	Arm 1	3/5 (60.0%)	0/5 (0%)	2/5 (40.0%)
	Arm 2	3/4 (75.0%)	0/4 (0%)	1/4 (25.0%)
	Arm 3	11/13 (84.6%)	1/13 (7.7%)	1/13 (7.7%)
	Total	17/22 (77.3%)	1/22 (4.5%)	4/22 (18.2%)
HVLT-R B	Arm 1	4/5 (80.0%)	0/5 (0%)	1/5 (20.0%)
	Arm 2	1/4 (25.0%)	1/4 (25.0%)	2/4 (50.0%)
	Arm 3	8/13 (61.5%)	1/13 (7.7%)	4/13 (30.8%)
	Total	13/22 (59.1%)	2/22 (9.1%)	7/22 (31.8%)
HVLT-R C	Arm 1	4/5 (80%)	0/5 (0%)	1/5 (20.0%)
	Arm 2	3/4 (75%)	0/4 (0%)	1/4 (25.0%)
	Arm 3	5/13(38.5%)	5/13 (38.5%)	3/13 (23.1%)
	Total	12/22 (54.5%)	5/22 (22.7%)	5/22 (22.7%)
TMTA	Arm 1	4/5(80.0%)	0/5 (0%)	1/5 (20.0%)
	Arm 2	3/4 (75.0%)	0/4 (0%)	1/4 (25.0%)
	Arm 3	7/11 (63.6%)	1/11 (9.1%)	3/11 (27.3%)
	Total	14/20 (70.0%)	1/20 (5.0%)	5/20 (25.0%)
TMTB	Arm 1	2/5 (40.0%)	0/5 (0%)	3/5 (60.0%)
	Arm 2	¾ (75.0%)	0/4 (0%)	1/4 (25.0%)
	Arm 3	9/12 (75.0%)	2/12 (16.7%)	1/12 (8.3%)
	Total	14/21 (66.7%)	2/21 (9.5%)	5/21 (23.8%)
COWA Total	Arm 1	5/5 (100.0%)	0/5 (0%)	0/5 (0%)
	Arm 2	1/4 (25.0%)	1/4 (25.0%)	2/4 (50.0%)
	Arm 3	13/13 (100.0%)	0/13 (0%)	0/13 (0%)
	Total	19/22 (86.4%)	1/22 (4.5%)	2/22 (9.1%)

Abbreviation: RCI, reliable change index.

## Discussion

Given the encouraging findings in the Phase I and II studies, and in particular in the GBM study, the proposed study was designed to further assess the efficacy and safety of ANG1005 in patients with HGG.^14,20^ The NCT01967810 clinical trial did not demonstrate significant efficacy in the heavily pretreated recurrent HGG patients. Yet, a dose of 600 mg/m^2^ was determined to be safe in this study. Furthermore, this trial highlights the importance of incorporating a companion biomarker/s that can identify responder patients to detect the signal of response.

Two Phase I, multicenter, sequential cohort, and dose-escalation studies were conducted with ANG1005 administered IV at doses ranging from 30 to 700 mg/m^2^ once every 3 weeks. The first study was in patients with primary brain tumors, and the second study was in patients with advanced solid tumors including breast cancer, lung cancer, ovarian cancer, melanoma, and others, in which most patients had brain metastases. ANG1005 was found to be tolerated at doses up to and including the MTD, which was determined to be 650 mg/m^2^ once every 3 weeks in both Phase I studies. Available data from prior studies^14,20,21^ showed higher tumor responses at 650 mg/m^2^ but better tolerability at 550 mg/m^2^ with reduced efficacy; therefore, 600 mg/m^2^ was selected to optimize safety and efficacy in this study.

In this Phase 2 study, ANG1005 demonstrated therapeutic activity in patients with recurrent grade III glioma in which one patient (3.6%) had a CR. Two patients (13.3%; total of 15 evaluable patients) had PR in the bevacizumab naïve recurrent GBM group (Arm 1). The clinical benefit rate (patients with SD or better) was 46.6% in Arm 1, 22.2% in Arm 2, and 53.6% in Arm 3, showing evidence of disease control in Arms 1 and 3. There was little evidence of sustained effect in Arm 2 (bevacizumab refractory recurrent GBM), a historically difficult-to-treat disease. Overall median PFS was 1.4 months (95% CI: 1.4, 2.1) across all arms. The median PFS was also similar across all treatment arms. Median OS was 13.4 months (95% CI: 3.4, 14.6) in Arm 1, 5.8 months (95% CI: 1.9, 9.7) in Arm 2, and 18.2 months (95% CI: 10.7, 35.3) in Arm 3. Based on the exposure–response analysis of all available data, a dose of 600 mg/m^2^ ANG1005 is the recommended optimal dose for future studies. The frequency and nature of the recorded AEs were consistent with the known safety profile of the study drug. The most commonly reported AEs were neutropenia (33%), alopecia (32%), and fatigue (30%). Based on RCI, neurocognitive function was stable or improved in 40–100% of patients in Arm 1, 50–75% in Arm 2, and 69–100% in Arm 3, across the battery of tests at the end of treatment. Overall, stable or improved neurocognitive function was seen more frequently than a decline following ANG1005 treatment.

Variable response to paclitaxel between patients is a well-known phenomenon for several cancers, with ~50% of patients being susceptible to this drug as a monotherapy. We recently reported endoplasmic reticulum translocon complex protein SSR3 as a predictive biomarker for glioma and breast cancer susceptibility to this drug.^22^ In a companion manuscript (Dmello et al., submitted to Neuro-Oncology Advances), we report an analysis of available tumor samples from this phase II trial (NCT01967810), where we evaluated the correlation with baseline SSR3 expression with OS on this cohort. This study showed that the patients with high SSR3 expression had a median OS of 18 months (95% CI 14.59, not applicable [NA]) compared to 9 months (95% CI 5.41, NA) in patients with low SSR3 expression (*P* = .2). No such trend was seen in the control cohort that did not receive ANG1005 treatment. This further suggests that upon overcoming the challenge posed by the BBB, effective delivery of paclitaxel into the brain can be efficacious in a subset of gliomas.

ANG1005 resulted in notable CNS antitumor activity in recurrent GBM and recurrent grade 3 glioma arms. Limited efficacy was seen in the bevacizumab-refractory population (arm 2), a historically difficult-to-treat populations where more clinical trials for our patients are warranted. Safety and tolerability of ANG1005 were consistent with the known AE profile of its derivative, paclitaxel. Similarly, delivery of albumin-bound paclitaxel to the brain using skull-implantable ultrasound and microbubbles for BBB opening was recently demonstrated to be safe and well tolerated.^23^

Notably, there were many limitations to this clinical trial. Most importantly, the results are considered preliminary due to the small number of subjects enrolled across diverse glioma histologies. A non-contemporary nature of tumor classification (WHO 2007 CNS tumor classification^16^) was used in categorizing patients in this study, and it is unclear if there is an efficacy signal in a specific tumor subtype based on WHO 2007, WHO 2016, and WHO 2021 modern classification of gliomas. Additionally, patients enrolled in the study were heavily pretreated with a median of 5 prior tumor-directed treatments. Efficacy may have been more robust if ANG1005 had been employed in earlier lines of treatment. Moreover, information related to prognostic factors (histology and grade, MGMT methylation, and prior response to first-line therapy) was not systematically collected for these patients. The imaging data was not collected on the patient that showed a CR, and the tumor tissue was also not available for contemporary classification. If a large ANG1005 clinical trial was to be conducted, we would recommend the use of a CRISPR screen-derived predictive biomarker to prospectively predict response in these patients.^22^ Importantly, ANG1005 was also shown to have efficacy in the breast cancer leptomeningeal disease setting^14^ and a large, randomized Phase 3 study is planned (NCT03613181).

## Conclusion

Although the pre-specified endpoints were not met, ANG1005 was shown to be safe in patients with recurrent HGG, including in patients with GBM who were bevacizumab refractory.

## Supplementary Material

vdae186_suppl_Supplementary_Figure

vdae186_suppl_Supplementary_Table

## Data Availability

Data will be made available upon reasonable request.
