# Supplementary material for: Phase II trial of blood–brain barrier permeable peptide-paclitaxel conjugate ANG1005 in patients with recurrent high-grade glioma
Source: Neurooncol Adv. 2024 Dec 14;6(1):vdae186. doi: 10.1093/noajnl/vdae186 (PMC11662161; doi:10.1093/noajnl/vdae186)
Supplement: vdae186_suppl_Supplementary_Figure [file vdae186_suppl_Supplementary_Figure.docx]

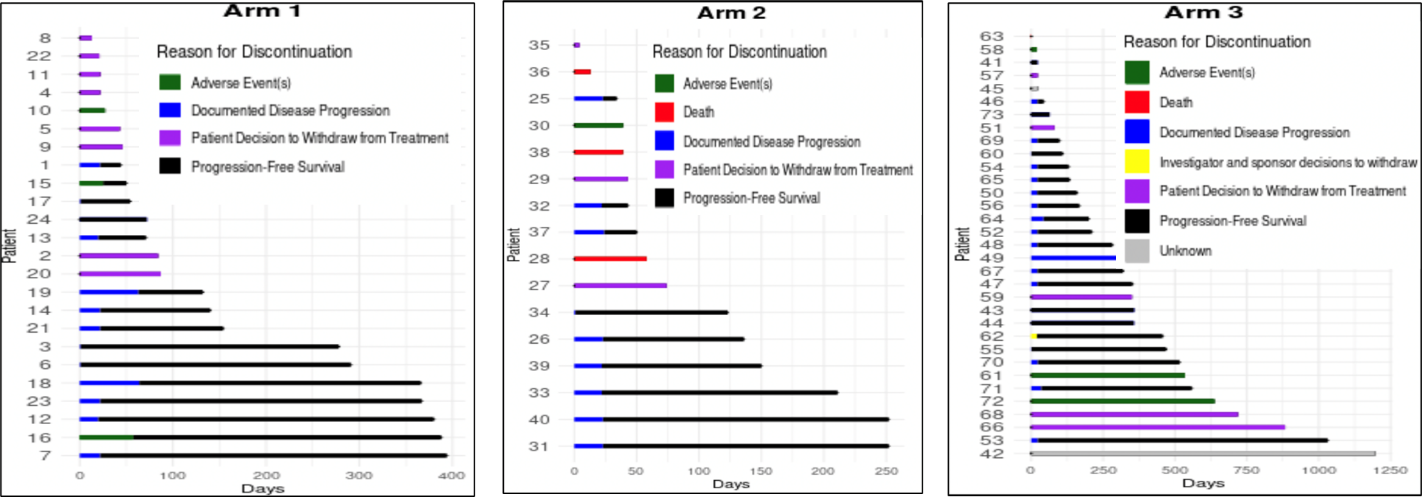


**Supplementary Figure 1: Swimmer plots showing time points of study treatment discontinuation in relation to PFS for all the three treatment Arms.** Subjects represented in entirely colored bars represent the time between the first dose and last follow-up as their PFS was unknown. For the bar first indicated by a colored segment, followed by a black segment, the colored segment represents the Time until Discontinuation corresponding to the reason for discontinuation by color, and the black segment indicates the PFS. If the bar only consists of black segment, the Time Until Discontinuation is unknown and the data represents only the PFS.
